# Understanding cancer genetic risk assessment motivations in a remote tailored risk communication and navigation intervention randomized controlled trial

**DOI:** 10.1080/21642850.2022.2150623

**Published:** 2022-12-09

**Authors:** Circe Gray Le Compte, Shou-En Lu, Julianne Ani, Jean McDougall, Scott T. Walters, Deborah Toppmeyer, Tawny W. Boyce, Antoinette Stroup, Lisa Paddock, Sherry Grumet, Yong Lin, Emily Heidt, Anita Y. Kinney

**Affiliations:** a Biobehavioral Cancer Health Equity Research Lab, Rutgers Cancer Institute of New Jersey, Rutgers, The State University of New Jersey, New Brunswick, NJ, USA; b Rutgers Environmental Epidemiology and Statistics, Rutgers University School of Public Health, Rutgers, The State University of New Jersey University, New Brunswick, NJ, USA; c Department of Internal Medicine, Division of Epidemiology, Biostatistics, and Preventive Medicine, University of New Mexico, Albuquerque, NM, USA; d Department of Health Behavior and Health Systems, University of North Texas Health Science Center, Fort Worth, TX, USA; e Stacy Goldstein Breast Cancer Center, LIFE Center, Medical Oncology, Rutgers Cancer Institute of New Jersey, Rutgers, The State University of New Jersey, New Brunswick, NJ, USA; f Biostatistics Shared Resource, UNM Comprehensive Cancer Center, University of New Mexico, Albuquerque, NM, USA; g New Jersey State Cancer Registry, Stroup Research Center, Rutgers Cancer Institute of New Jersey, Rutgers, The State University of New Jersey, New Brunswick, NJ, USA; h Cancer Surveillance Research Program, Rutgers Cancer Institute of New Jersey, Rutgers, The State University of New Jersey, New Brunswick, NJ, USA; i LIFE Center, Rutgers Cancer Institute of New Jersey, Rutgers, The State University of New Jersey, New Brunswick, NJ, USA; j Department of Biostatistics and Epidemiology, School of Public Health, Rutgers Cancer Institute of New Jersey, Rutgers, The State University of New Jersey University, New Brunswick, NJ, USA

**Keywords:** Genetic testing, genetic counseling, cancer, hereditary breast and ovarian cancer

## Abstract

**Background:**

National guidelines recommend cancer genetic risk assessment (CGRA) (i.e. genetic counseling prior to genetic testing) for women at increased risk for hereditary breast and ovarian cancer (HBOC). Less than one-half of eligible women obtain CGRA, leaving thousands of women and their family members without access to potentially life-saving cancer prevention interventions.

**Purpose:**

The Genetic Risk Assessment for Cancer Education and Empowerment Project (GRACE) addressed this translational gap, testing the efficacy of a tailored counseling and navigation (TCN) intervention vs. a targeted print brochure vs. usual care on CGRA intentions. Selected behavioral variables were theorized to mediate CGRA intentions.

**Methods:**

Breast and ovarian cancer survivors meeting criteria for guideline-based CGRA were recruited from three state cancer registries (*N *= 654), completed a baseline survey, and were randomized. TCN and targeted print arms received the brochure; TCN also participated in a tailored, telephone-based decision coaching and navigation session grounded in the Extended Parallel Process Model and Ottawa Decision Support Framework. Participants completed a one-month assessment. Logistic regression was used to compare the rate of CGRA intentions. CGRA intentions and theorized mediator scores (continuous level variables) were calculated using mixed model analysis.

**Results:**

CGRA intentions increased for TCN (53.2%) vs. targeted print (26.7%) (OR = 3.129; 95% CI: 2.028, 4.827, *p* < .0001) and TCN vs. usual care (23.1%) (OR = 3.778, CI: 2.422, 5.894, *p* < .0001). Perceived risk (*p* = 0.023) and self-efficacy (*p* = 0.035) mediated CGRA intentions in TCN.

**Conclusions:**

Improvements in CGRA intentions and theorized mediators support the use of a tailored communication intervention among women at increased HBOC risk. (Clinicaltrials.gov: NCT03326713.)

**Trial registration:** ClinicalTrials.gov identifier: NCT03326713.

## Background

Pathogenic variants in cancer predisposition genes account for up to 20% of all cancers (Finch et al., 2014; Litton et al., 2018; National Comprehensive Cancer Network, 2021; Robson et al., 2017). *BRCA1* and *BRCA2* carry a cumulative breast cancer risk of 69% and 44%, and a cumulative ovarian cancer risk of 44% and 17%, respectively, for women (individuals assigned female at birth) up to 80 years old (Kuchenbaecker et al., 2017). These mutations are associated with an increased risk of second, hereditary breast and ovarian cancer (HBOC) among breast and/or ovarian cancer survivors, and primary cancers, such as breast, ovarian, pancreatic, and prostate cancers, among their biological relatives (Childers et al., 2017; Ji et al., 2020; Tung et al., 2016; Wood et al., 2012). For two decades, National Comprehensive Cancer Network guidelines have recommended cancer genetic risk assessment (CGRA), which encompasses genetic counseling prior to genetic testing, for all women diagnosed with epithelial ovarian cancer and/or high-risk breast cancers or otherwise considered at risk for HBOC (Daly et al., 2017; Kataoka, 2021; National Comprehensive Cancer Network, 2021; US Preventive Services Task Force, 2019). This group represents a subgroup of individuals at high risk of breast, ovarian, and hereditary cancers (Daly et al., 2017; Kataoka, 2021; US Preventive Services Task Force, 2019). Research has shown that genetic counseling and testing offer breast and ovarian cancer survivors opportunities beyond standard medical care to access cancer prevention services for secondary cancers (Cragun et al., 2017). Individuals at increased risk may consider enhanced screening, prophylactic surgeries, and other actions to reduce their cancer risks or improve early detection. This can be particularly important for those who did not understand their risk for hereditary and secondary cancers or believed they could not do anything to prevent these cancers (Cragun et al., 2017; Smith-Uffen et al., 2021). Additionally, women who obtain genetic testing may share their results with close biological relatives, serving as informal educators/advocates, informing them of possible cancer risks and linking them to information and resources (Carpenter & Sherbino, 2010).

Despite the benefits of CGRA, studies indicate that less than 50% of eligible breast or ovarian cancer survivors seek genetic counseling or testing, leaving thousands of women and their family members unaware of their HBOC risk and without access to potentially life-saving cancer prevention resources (Dwyer et al., 2021; Kurian et al., 2017; Nelson et al., 2019). Black, indigenous, and persons of color (BIPOC), rural dwellers, and those with low health literacy have even lower rates of CGRA, which may contribute to their disparate burden of breast and ovarian cancer (Delikurt et al., 2015; Kurian et al., 2017; Smith-Uffen et al., 2021). Women at increased HBOC risk may face significant barriers to CGRA, such as lack of provider referral (Delikurt et al., 2015; Kurian et al., 2017; Smith-Uffen et al., 2021), as well as psychosocial factors, including fatalism and cancer worry (Cragun et al., 2019; Gómez-Trillos et al., 2020; Hann et al., 2017; Kinney et al., 2010; Komenaka et al., 2016; Sutton et al., 2019).

The Genetic Risk Assessment for Cancer Education and Empowerment (GRACE) Project sought to eliminate the translational gap among women at increased HBOC risk. GRACE developed a tailored counseling and navigation intervention (TCN) based on research from the Family Colorectal Cancer Awareness and Risk Education Project. This two-group randomized trial found that participants who received the study’s tailored intervention had nearly three times the colonoscopy uptake as those who received an educational brochure (Birmingham et al., 2015; Boonyasiriwat et al., 2014; Brumbach et al., 2017). GRACE expanded this efficacious intervention with motivational psychoeducation and navigation components to increase CGRA uptake. The approach differentiated TCN from standard genetic counseling, which focuses on clinical risk assessments, genetic education, and discussion of genetic risk and the benefits, limitations, and risks of genetic testing to promote informed decision-making in a non-directive manner. In contrast, TCN’s tailored education and motivation strategy encouraged participants to formulate a concrete plan to obtain pre-test genetic counseling by a clinical cancer genetic risk specialist (e.g. a genetic counselor). TCN was delivered remotely by telephone, an approach shown to enhance reach and participant engagement (Anderson-Lewis et al., 2018; Rees et al., 2018; Steffen et al., 2015). We compared TCN to a targeted print arm and a usual care arm.

## Theoretical framework

Targeted print materials, which include resources produced for specific audiences, offer an efficient, low-cost public health strategy to disseminate health information (Sabatino et al., 2012). However, research indicates that theory-based psychosocial interventions featuring tailored (personalized) messages more effectively address behavioral determinants and motivate recommended health behaviors (Acharya et al., 2021; Flores et al., 2017; Sabatino et al., 2012). Therefore, TCN employed tailored risk communication messages targeting behavioral constructs drawn from an integrated theoretical framework (Hoefel et al., 2020; Schwarzer et al., 2008; Witte, 1992), featuring the Extended Parallel Process Model. This model posits that risk communication messages arouse (1) *threat appraisals*, individuals’ perceived susceptibility to HBOC and perceived severity of HBOC’s potential harm, and (2) *efficacy appraisals*, individuals’ beliefs in an intervention’s *response efficacy* to a threat (CGRA’s utility to reduce cancer risk) and their self-efficacy in participating in preventive health behavior (confidence in obtaining CGRA) (Witte, 1992). While low perceived threat could encourage deferral of recommended health behaviors, high perceived threat can engender maladaptive inaction and avoidance (Maloney et al., 2011; Witte & Allen, 2000). TCN aimed to increase perceived threat and susceptibility to HBOC while building their belief in CGRA’s response efficacy and their own self-efficacy (Maloney et al., 2011; Witte & Allen, 2000). The Health Action Process Approach provided tailored planning and support constructs to bridge the gap between CGRA intentions and uptake (Schwarzer & Hamilton, 2020; Zhang et al., 2019). The Ottawa Decision Support Framework addressed cognitive factors to motivate informed decision-making (Stacey et al., 2020). Cancer education specialists delivered TCN messages using motivational interviewing, an evidence-based counseling style eliciting motivation and commitment to change (Miller & Rollnick, 2012).

**Figure 1.**
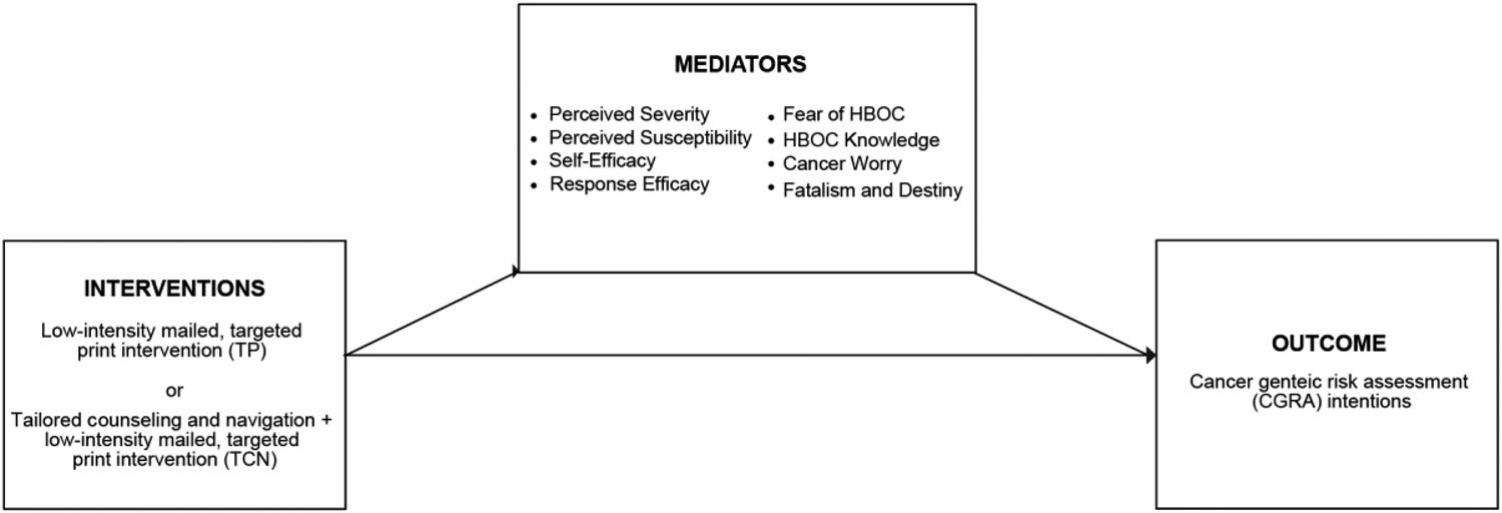
Mediation analysis models: theorized mediators of GRACE interventions on intentions to seek CGRA.

As shown in Figure 1, we hypothesized that perceived susceptibility, perceived severity, perceived self-efficacy, response efficacy, HBOC knowledge, fear of HBOC, cancer worry, and fatalism and destiny would mediate CGRA intentions from baseline to the one-month follow-up. As a result, CGRA intentions would be higher among TCN participants than the targeted print and usual care arms at the one-month follow-up. (We surmised that perceived severity would remain constant due to near-universal belief in cancer’s potential harm.)

## Materials and methods

**Figure 2.**
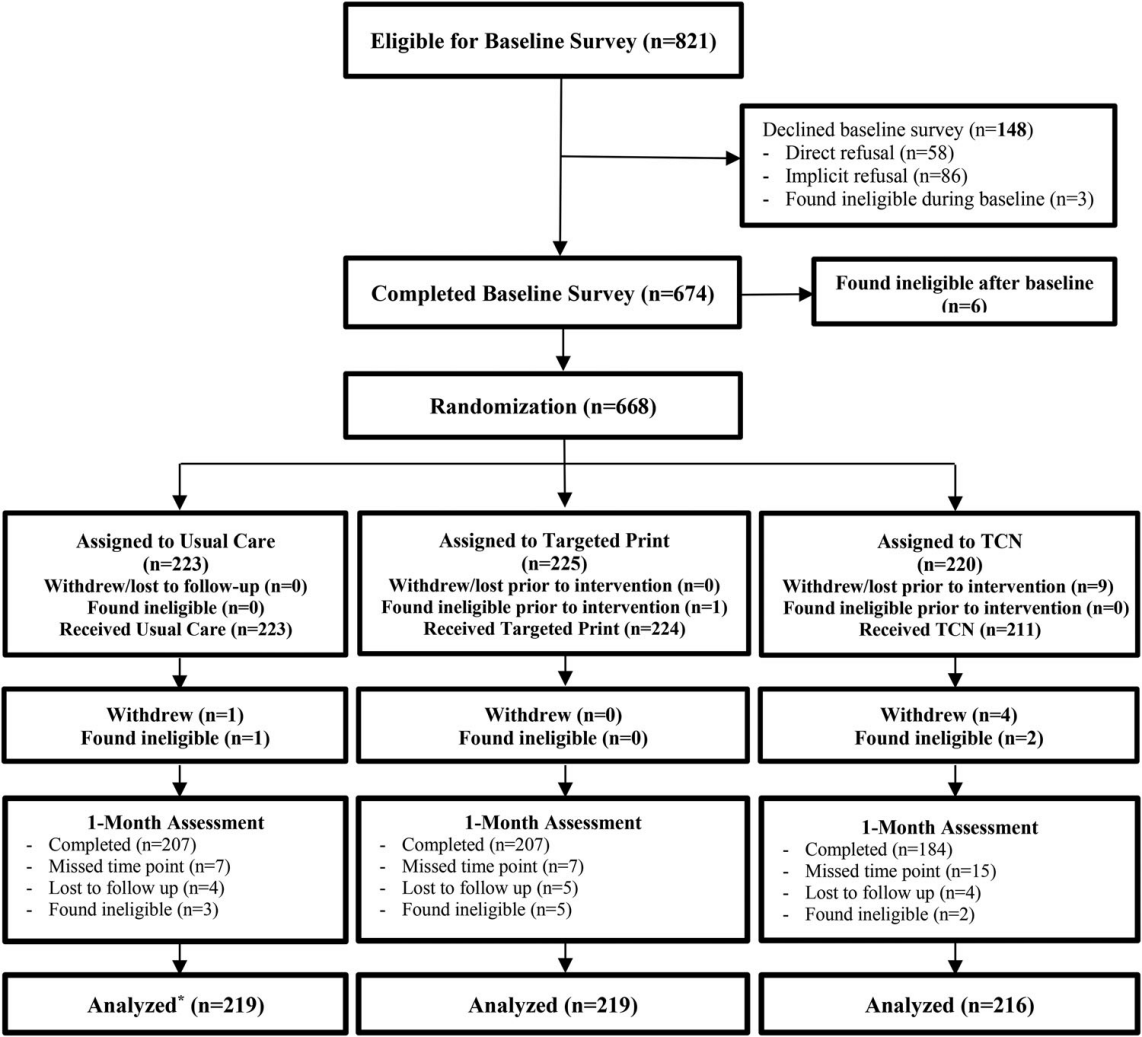
CONSORT diagram of the GRACE project randomized-control trial from baseline to the one-month follow-up. Note: * In the intent-to-treat analysis, only participants found ineligible were excluded.

***Study Design:*** GRACE, a three-arm superiority trial, tested TCN vs. a mailed, targeted print brochure vs. usual care. CGRA uptake was hypothesized to be highest in TCN vs. the targeted print vs. the usual care arms at 6 and 12 months (Kinney et al., 2018). The trial followed the recommended standards of the extended Consolidated Standards of Reporting Trials (CONSORT) statement for parallel group, non-pharmacologic randomized trials (Figure 2), and was approved by the Institutional Review Boards of each participating institution (Boutron et al., 2017). All participants enrolled in the study provided informed consent. Study data were collected and managed in REDCap (Research Electronic Data Capture), a secure, web-based data capture tool hosted by the University of New Mexico and the Rutgers Cancer Institute of New Jersey (Harris et al., 2009). This study used data collected from baseline and the one-month follow-up, examining whether specified theoretical targets mediated CGRA intentions.

***Study Participants:*** We recruited 654 participants using Traceback, a population-based approach to identifying and engaging individuals at increased risk for HBOC, through three statewide cancer registries: Colorado Central Cancer Registry, New Jersey State Cancer Registry, and New Mexico Tumor Registry (Moss et al., 2018; Samimi et al., 2017). According to state and federal laws, these registries work with hospitals, clinics, laboratories, surgical centers, radiology departments, and other entities to collect and maintain data on cancer cases, tracking statewide trends in cancer incidence (Kumar et al., 2020; Ryerson et. al., 2015).

To be eligible for GRACE, individuals could not have had CGRA, and must be Colorado, New Mexico, or New Jersey residents; ≥ 21 years of age; assigned female sex at birth; fluent in English or Spanish; not in hospice; and diagnosed with at least one guideline-based CGRA condition: breast cancer (≤ age 50); triple-negative breast cancer (≤ age 60); ovarian, fallopian, or peritoneal cancer (diagnosed at any age); or ≥ two primary breast cancers.

***Screening and Random Assignment:*** Eligible cancer survivors received a letter in English and Spanish describing GRACE, along with a recruitment brochure, contact information form, and an opt-out form. Those who did not return the opt-out form within three weeks were contacted by telephone. Eligible, interested women provided informed consent and completed surveys online or by telephone based on their preferences and circumstances (e.g. access to a computer). After completing the baseline survey, participants were randomized to TCN, targeted print, or usual care using a computer-generated random number list with a block size of nine.

***Study Arms:*** Participants in usual care, the control arm, were directed to continue their current course of health care. Those randomized to targeted print received a letter about their assignment and a copy of the educational brochure. The brochure’s content aligns with the Extended Parallel Process Model and provides an overview of HBOC, its risk factors, how CGRA can help mitigate HBOC risk, where to obtain CGRA, genetic confidentiality protections provided by the Genetic Information Nondiscrimination Act (GINA), and geographically targeted resources and services. Most of the content is written at a <6th grade readability level, though clinical terms integral to the content increase its readability level overall to an 8th grade level. The brochure and visual aids described below feature culturally appropriate content and images informed by GRACE’s theoretical framework and feedback gleaned from our formative research (Kinney et al., 2018). The formative research encompassed focus groups with 13 Hispanic women, and a survey in 2014–2015 of 213 high-risk Hispanic and non-Hispanic cancer survivors who had obtained genetic testing and received uninformative results, as well as their close biological relatives (Kinney et al., 2018). Survey participants offered guidance on the brochure and visual aids in 2015–2016 through Learner Verification and Revision (LV&R), a research approach intended to ensure that educational messages support intended awareness and adoption of health behaviors (Chavarria et al., 2021; Kinney et al., 2018).

TCN participants received a letter detailing their study group assignment, the brochure, and a sealed envelope of visual aids. Within two weeks of receiving these materials (and approximately two weeks before the one-month follow-up survey), TCN participants engaged in a 30–45 min tailored, stepped psychoeducational counseling session with a cancer education specialist (coach). Coaches received training from a motivational interviewing expert who periodically reviewed session tapes to ensure intervention fidelity and provide feedback on approach and participant engagement (Kinney et al., 2018). Leveraging a neutral, non-judgmental style, coaches tailored session scripts based on baseline survey responses, including content matching on theoretical and social determinants. Coaches further personalized sessions based on feedback, engendering trust and encouraging participants to articulate concerns, such as fear of HBOC. At the start of the discussion, coaches asked participants to open the visual aids, which included graphic representations of key statistics about HBOC risk and graphical tools to gauge their beliefs about CGRA’s importance and their readiness to schedule CGRA in the next 6 months. Below is a synopsis of the intervention steps, described in detail in another publication (Kinney et al., 2018):

***Step 1***. Coaches built rapport with participants, asking them to describe their personal and familial cancer histories and current cancer prevention approaches. Participants then opened their packet of visual aids, which served as touchstones during the ensuing discussion.
***Step 2***. Coaches discussed hereditary cancer, CGRA, and GINA, using metaphors to explain complex concepts (e.g. comparing genetic mutations to changes in a family recipe), asking participants to share their understanding and knowledge on a topic, and providing clarification as needed.
***Step 3***. Coaches explored participants’ perceived HBOC susceptibility and severity, eliciting change talk about CGRA to mitigate defensive responses, such as message rejection and fear.
***Step 4***. Response efficacy was similarly addressed, with participants responding to information about CGRA’s utility in mitigating the risk of HBOC and other cancers.
***Step 5***. Coaches helped participants reflect on personal facilitators and barriers to CGRA uptake, such as insurance status and access to genetic testing. On a scale from 1 (low) to 10 (high), participants rated: (1) how important they believed it was to obtain CGRA in the next six months, and (2) how ready they felt to make a CGRA appointment in the next six months.
***Step 6***. Participants set up an action plan with their coach to access CGRA.
***Step 7***. Coaches reviewed the session and action plan with participants, informing them that a letter summarizing their session would be mailed to them after the call. Participants also were invited to have a copy of the summary letter sent to the provider.
***Step 8***. Coaches told participants that an Action Plan Reminder Card about their CGRA plan would arrive in the mail six weeks after the session.
***Step 9***. Coaches scheduled a follow-up call with participants to take place seven weeks after the session. During that call, coaches confirmed participants’ receipt of the Action Plan Reminder Card and determined if additional navigation assistance was required (Kinney et al., 2018).

### Data collection and measures

Prior to randomization, participants completed a baseline survey, which collected information on the theorized mediator variables and sociodemographic factors (Kinney et al., 2018). Participants then engaged in study arm activities and completed the one-month follow-up survey. We assessed whether the theorized constructs (listed below) mediated the relationship between the TCN, targeted print, and usual care arms and the outcome, CGRA intentions. ‘CGRA intentions’ was measured using the question: ‘How likely do you think it is that you will undergo cancer genetic risk assessment for hereditary breast and ovarian cancer within the next 6 months?’ (CGRA intentions represent an interim measure. CGRA uptake was measured at the 6-month and 12-month time points, and will be assessed in future studies.) Participants answered using a Likert scale (1 = Not at All Likely to 5 = Extremely Likely). The outcome was dichotomized as a yes/no item to assess the odds of CGRA intentions (no = 1, 2, or 3; yes = 4 or 5), and treated as a continuous measure for mixed model analysis. We customized the measures to reflect an HBOC focus, and calculated Cronbach’s alpha scores at baseline and the one-month follow-up as follows:

- *Perceived susceptibility* (baseline *α* = 0.89, one-month follow-up *α* = 0.89) and *perceived severity* (baseline *α* = 0.84, one-month follow-up *α* = 0.82) subscales, adapted from the Risk Behavior Diagnosis Scale (Witte et al., 2001), each contained four items, scored 1–5. Total scores for each scale ranged from 4–20; higher scores indicated greater belief in HBOC threat and impact on health.
- *Self-efficacy* (baseline *α* = 0.84, one-month follow-up *α* = 0.84) and *response efficacy* (baseline *α* = 0.92, one-month follow-up *α* = 0.93), also Risk Behavior Diagnosis Scale subscales; each contained four items, scored 1–5. Total scores for each scale ranged from 4–20; higher scores indicated greater belief in CGRA and one’s ability to obtain it, respectively (Witte et al., 2001).
- *HBOC knowledge* (baseline *α* = 0.89, one-month follow-up *α* = 0.86), adapted from the National Center for Human Genome Research Questionnaire, tested participants’ understanding of HBOC. Participants’ answers to each of the scale’s eleven items received scores of either 0 (incorrect) or 1 (correct). Higher total scores indicated greater HBOC knowledge (Langer et al., 2017).
- *Fear of HBOC* (baseline *α* = 0.94, one-month follow-up *α* = 0.94), derived from the Cancer Risk Beliefs Scale’s Negative Affect in Risk subscale, captured participants’ perceived HBOC risk. The scale’s six items were scored from 1 (strongly disagree) to 4 (strongly disagree). Scale scores were added together and the mean taken. Higher mean scores indicated greater HBOC fear (Baser et al., 2019).
- *Cancer worry* measured two domains: frequency and intensity of cancer worry (Caruso et al., 2018). Cancer worry–frequency was measured with a single item, and cancer worry–intensity was calculated from the sum of two items (baseline *α* = 0.93, one-month follow-up *α* = 0.92). Higher scores signified greater intensity of cancer worry–frequency and/or cancer worry–intensity. All cancer worry items were scored on a Likert scale ranging from 1 (never) to 5 (all the time). (Cronbach’s alpha was not calculated for cancer worry–frequency since the construct contained only one item.)
- The *fatalism and destiny* scale (baseline *α* = 0.55, one-month follow-up *α* = 0.57) measured thoughts and feelings about cancer prevention. The scale’s three items were scored on a Likert scale [strongly disagree (1) to strongly agree (5)] and, due to their weak correlation, analyzed individually. Higher scores indicated greater pessimism, helplessness, and thoughts of death (Shen et al., 2009).

## Analysis

Biostatisticians blinded to study arm assignment performed an intent-to-treat analysis using SAS v9.4 (SAS Institute, Inc., Cary, North Carolina), and RMediation package available in R (Tofighi & MacKinnon, 2011). Summary statistics of baseline patient sociodemographic variables were compared among treatment arms using ANOVA and χ^2^ tests. Pearson correlation coefficients were calculated to discern potential correlations between the selected theorized mediator variables (Evans, 1996). We calculated Cronbach’s alpha to ensure internal consistency (the extent to which their respective items measured the same construct) across measures (Tavakol & Dennick, 2011) at baseline and the one-month follow-up (reported in methods).

Logistic regression was used to compare the percentage of CGRA intentions reported at the one-month follow-up between study arms; odds ratios and 95% confidence intervals (CIs) were calculated. We leveraged mixed model analysis to assess longitudinal between-treatment-arm and pre-to-post-differences in the scores for CGRA intentions and each theorized mediator variable. (The approach also accounted for repeated measures in our study design, as well as the 95% CIs, using linear contrasts.) We then performed mediation analysis to assess whether improvement in CGRA intentions was mediated by pre-to-post changes of the theorized meditator variables. (The methods and results of the between-group mediation are reported in Supplemental Materials section.)

Due to the significant improvement in CGRA intentions for TCN compared to the other study arms, we studied the mediational relationships for CGRA intentions in TCN using the within-group analysis. We first conducted a single variable mediation analysis, in which only one theorized mediator was introduced into the model to assess its indirect effects on CGRA intentions. We followed this approach with multivariable mediation analysis, in which the theorized mediators were introduced into the model simultaneously to assess their indirect effects on the outcome. Specifically, we fitted the following models: Model 1.1: *Y*_it _= *c*_0i _+ *c*_1_time_it _+ *e*_1it_; Model 1.2: *M*_it _= *a*_0i _+ *a*_1_time_it _+ *e*_2it_; and Model 1.3: *Y*_it _= *b*_0i _+ *b*_1_time_it _+ *b*_2_*M*_it_ + *e*_3it._ In these models, *Y_ij_* and *M_ij_* represented CGRA intentions and mediator of subject *i* at time *j* (*j* = 0 for baseline and *j* = 1 for one-month post-intervention), respectively. We tested H_0_: *a_1_b_2 _*= 0 using the confidence interval (CI) approach (MacKinnon et al., 2007). If 0 was not included in 95% CI, then we rejected H_0_ and established the mediation relationship. The percentage of indirect effect for significant theoretical intervention variables was calculated as P_M _= *a_1_b_2_/c_1_* (MacKinnon et al., 2007).

## Statistical power

In the original study protocol, GRACE was powered to discern between-group comparisons to assess the study’s primary outcome: CGRA uptake at 6 months. We conducted a *post hoc* power analysis to assess between and within-group differences from baseline to the one-month follow-up. Cohen’s *d* measures, which here reflect standardized effect sizes derived from the difference in hypothesized mediators for baseline and the follow-up assessment, are reported for between and within-group analyses, in Table 4.

For the between-group analysis, the sample size for each study arm, TCN (*n* = 216), targeted print (*n* = 219), and usual care (*n* = 219) (reported in Table 1) powered the study at 80% to detect a minimal pairwise between-group difference of Cohen's *d *= 0.27 (*α* = 0.050, 2-sided), and Cohen’s *d *= 0.31 (*α* = 0.017, after Bonferroni adjustment for three between-group comparisons, 2-sided). In the within-group analysis, the sample size for each study arm provided power at 80% to detect within-group differences between Cohen's *d *= 0.19 (*α* = 0.05) and Cohen’s *d *= 0.22 (*α* = 0.017, after Bonferroni adjustment for three within-group comparisons).

**Table 1.** Sociodemographic characteristics of participants by study arm.

Study arm	All (*N* = 654) *n* (%)	Usual Care (*N* = 219) *n* (%)	Targeted Print (*N* = 219) *n* (%)	TCN (*N* = 216) *n* (%)	*p*-value
Age (Mean, SD)	61.3 (10.1)	61.2 (9.9)	61.3 (10.0)	61.4 (10.7)	0.9939
Years since diagnosis (Mean, SD)	11.1 (7.6)	11.2 (7.6)	10.9 (7.5)	11.3 (7.7)	0.8949
Self-reported race/ethnicity					0.5313
Hispanic/Latina Persons	165 (26.7)	57 (27.4)	48 (23.4)	60 (29.3)	
Non-Hispanic White Persons	389 (62.9)	134 (64.4)	133 (64.9)	122 (59.5)	
Non-Hispanic Black Persons	39 (6.3)	9 (4.3)	17 (8.3)	13 (6.3)	
Non-Hispanic Asian Persons	25 (4.0)	8 (3.8)	7 (3.4)	10 (4.9)	
Other Persons	36	11	14	11	
Self-reported Ashkenazi Jewish ancestry					0.5637
No	606 (97.1)	202 (97.1)	206 (96.3)	198 (98.0)	
Yes	18 (2.9)	6 (2.9)	8 (3.7)	4 (2.0)	
Missing	30	11	5	14	
Marital Status					0.5189
Single/Divorced/Separated/Widowed	255 (39.2)	79 (36.4)	91 (41.7)	85 (39.5)	
Married/Domestic Partnership	395 (60.8)	138 (63.6)	127 (58.3)	130 (60.5)	
Missing	4	2	1	1	
Education level					0.3726
<High School/High School Grad/GED	118 (18.3)	44 (20.4)	41 (18.9)	33 (15.6)	
Some college, Assoc. Degree, or Voc. School	233 (36.1)	83 (38.4)	70 (32.3)	80 (37.7)	
Bachelor’s Degree or higher	294 (45.6)	89 (41.2)	106 (48.8)	99 (46.7)	
Missing	9	3	2	4	
Annual household income, $					0.9539
<$30,000	143 (24.8)	52 (27.1)	45 (23.2)	46 (24.2)	
$30,000–$49,999	103 (17.9)	36 (18.8)	35 (18.0)	32 (16.8)	
$50,000–$69,999	86 (14.9)	29 (15.1)	29 (14.9)	28 (14.7)	
$70,000 or more	244 (42.4)	75 (39.1)	85 (43.8)	84 (44.2)	
Missing	78	27	25	26	
Healthy Literacy Level*					0.7290
Adequate (<9)	42 (6.5)	10 (4.7)	14 (6.5)	17 (8.0)	
Marginal (5 ≥ 9)	189 (29.5)	65 (30.5)	62 (28.8)	62 (29.2)	
Inadequate (≥5)	138 (64.8)	133 (62.7)	139 (64.7)	198 (92.1)	
Missing	4	2	1	1	
Rural vs. urban residence**					0.1058
Urban	539 (82.7)	189 (87.1)	177 (80.8)	173 (80.1)	
Rural	113 (17.3)	28 (12.9)	42 (19.2)	43 (19.9)	
Missing	2	2			
Has health insurance					0.8628
No	13 (2.3)	5 (2.5)	5 (2.5)	3 (1.8)	
Yes	559 (97.7)	196 (97.5)	195 (97.5)	168 (98.2)	
Missing	82	18	19	45	
Has a personal health care provider					0.9493
No	26 (4.1)	9 (4.2)	8 (3.7)	9 (4.2)	
Yes	615 (95.9)	204 (95.8)	208 (96.3)	203 (95.8)	
Missing	13	6	3	4	
Cancer Site					0.4002
Ovarian	94 (14.7)	36 (16.9)	32 (14.8)	26 (12.3)	
Breast	547 (85.3)	177 (83.1)	184 (85.2)	186 (87.7)	
Missing	13	6	3	4	
Number of first- (FDR) and second-degree relatives (SDR) with breast or ovarian cancer					0.8375
0 FDR and 0 SDR	420 (64.4)	136 (62.7)	145 (66.2)	139 (64.4)	
1 FDR or 1 SDR	131 (20.1)	49 (22.6)	40 (18.3)	42 (19.4)	
2 or more FDR/SDR	101 (15.5)	32 (14.7)	34 (15.5)	35 (16.2)	
Missing	2	2			
Years since diagnosis					0.6240
<5	166 (25.6)	52 (24.1)	59 (27.2)	55 (25.5)	
5 to <10	158 (24.3)	60 (27.8)	46 (21.2)	52 (24.1)	
≥10	325 (50.1)	104 (48.1)	112 (51.6)	109 (50.5)	
Missing	5	3	2		
Survey Mode at Baseline					0.1542
Online	420 (65.5)	130 (61.0)	151 (69.9)	139 (65.6)	
Telephone	221 (34.5)	83 (39.0)	65 (30.1)	73 (34.4)	
Survey Mode at One-Month					0.2709
Online	410 (66.9)	133 (63.6)	151 (70.9)	126 (66.0)	
Telephone	203 (33.1)	76 (36.4)	62 (29.1)	65 (34.0)	

* Short Test of Functional Health Literacy in Adults (STOHFLA) (Chew et al., 2004) .

** Rural or urban residence was based on Rural-Urban Commuting Area (RUCA) codes at the zip code level. RUCA codes were developed by the University of Washington Rural Health Research Center and the United States Department of Agriculture Economic Research Service (ERS), with the support of the Health Resource and Service Administration’s Office of Rural Health Policy and the ERS, using standard Census Bureau urbanized area and urban cluster definitions in combination with work commuting data to characterize census tracts and then zip codes. The 10 RUCA categories were aggregated into urban (1–3) and rural (4–10), as recommended by the WWAMI (Washington, Wyoming, Alaska, Montana, and Idaho) Rural Health Research Center (U.S. Department of Agriculture 2000).

For the between-group analysis, our power analysis showed that in comparing CGRA intentions, the effect sizes for TCN vs. targeted print (Cohen’s *d *= 0.62) and TCN vs. usual care (Cohen’s *d *= 0.64) exceeded the minimal detectable effect sizes (Cohen’s *d *= 0.27∼0.31). For the hypothesized mediators, only the effect size of cancer worry–intensity (TCN vs. targeted print: Cohen’s *d *= 0.34) exceeded the minimal detectable difference. These results suggested that our study generally lacked power in most between-group differences shown in the hypothesized mediators. As such, in this relatively large study, we could not establish the mediational relationships as hypothesized (shown in Supplemental Table 1), prompting us to focus on within-group differences.

We observed substantial increases in CGRA intentions in the TCN study arm from baseline to the one-month follow-up. Thus, we performed a mediation analysis to study what potential mediators contributed to this improvement. Specifically, for the within-group analysis, we observed small effect sizes for targeted print (Cohen’s *d *= 0.26) and usual care (Cohen’s *d *= 0.23), and a large effect size for TCN (Cohen’s *d *= 0.76). We also noted small effect sizes in TCN for perceived susceptibility (Cohen’s *d *= 0.25), perceived severity (Cohen’s *d *= 0.22), and self-efficacy (Cohen’s *d *= 0.17). In both the single-variable and multivariable mediation analyses, reported in Tables 5 and 6, respectively, we observed significant increases in perceived susceptibility and self-efficacy among TCN participants, which contributed indirectly to TCN’s effects on CGRA intentions.

## Results

The GRACE study team attempted to contact 4451 women with breast and/or ovarian cancer who were identified through the New Mexico, Colorado, and New Jersey cancer registries and referred to the study as potentially eligible. We were able to contact 2810 women; 489 declined participation and 1500 were deemed ineligible primarily because they had prior genetic counseling and/or testing. Of the 821 individuals who were screened and met the eligibility criteria, 654 (79.7%) enrolled. The retention rate at the one-month assessment was 91.3%. Nearly two-thirds of participants (65.5%) completed the surveys online rather than by telephone (34.5%). Chi-square tests for clinical and sociodemographic characteristics (Table 1), indicated a balanced distribution across study arms. We also observed highly acceptable Cronbach’s alphas for the theorized mediators (shared in Methods), with only fatalism and destiny scoring below the acceptable threshold of 0.70 at baseline (0.55) and the one-month follow-up (0.57) (Evans, 1996). In our correlation analysis, shown in Table 2, we observed primarily very weak (*r* = 0.00–0.19) to weak (*r* = 0.20–0.39) correlations among our theorized mediators. Exceptions included a moderate correlation (*r* = 0.40–0.59) between fear of HBOC and cancer worry–frequency (*r* = 0.437) and fear of HBOC and cancer worry–intensity (*r* = 0.584) and a strong correlation (*r* = 0.60–0.79) between cancer worry–intensity and cancer worry–frequency.

**Table 2.** Correlations matrix of theorized mediator variables.

Mediation Variables	1 Perceived Severity	2 Perceived Susceptibility	3 Response Efficacy	4 Self-Efficacy	5 HBOC Knowledge	6 Fear of HBOC	7 Cancer Worry–Frequency	8 Cancer Worry–Intensity	9 Fatalism and Destiny
1 Perceived Severity	1.000	0.212***	0.390***	0.090*	0.139***	0.233***	0.104**	0.152***	0.014
2 Perceived Susceptibility		1.000	0.367***	0.205***	0.113**	0.365***	0.148***	0.170***	0.036
3 Response Efficacy			1.000	0.389***	0.123**	0.302**	0.140***	0.187***	−0.031
4 Self-Efficacy				1.000	0.051	0.098*	0.033	0.077*	−0.050
5 HBOC Knowledge					1.000	0.134***	−0.016	0.023	−0.104**
6 Fear of HBOC						1.000	0.437***	0.584***	0.256***
7 Cancer Worry–Frequency							1.000	0.733***	0.184***
8 Cancer Worry–Intensity								1.000	0.264***
9 Fatalism and Destiny									1.000

*p < .05.

**p < .01.

***p < .001.

In the logistic regression, reported in Table 3, we observed a significant increase in odds of CGRA intentions at the one-month assessment for both TCN vs. targeted print (OR = 3.129; CI: 2.028, 4.827; *p* < 0.0001) and TCN vs. usual care (OR = 3.778; CI: 2.422, 5.894; *p* < 0.0001). We did not observe a significant difference between targeted print vs. usual care. For the mixed model analysis, we compared differences in the scores of CGRA intentions (continuous level variables) and the theorized mediators between study arms. As reported in Table 4, we observed greater improvement in CGRA intentions in TCN vs. targeted print (0.642, *p* < 0.001, CI: 0.315, 0.969) and TCN vs. usual care (0.694, *p* < 0.001, CI: 0.367, 1.021). Among the mediators, we observed significant pre-to-post increases in TCN for the theoretical constructs, perceived susceptibility (0.773, *p* = 0.023, CI: 0.109, 1.437) and perceived self-efficacy (0.666, *p* = 0.035, CI: 0.049, 1.283). Differences in the scores of the theorized mediators between study arms also are reported in Table 4.

**Table 3.** CGRA intentions at baseline and the one-month follow-up.

**3a. CGRA Intentions at Baseline**				**CGRA Intentions = Yes**							
			* *	**Total**		**TCN**		**Targeted Print**		**Usual Care**	
**Study Arms Comparisons**	**Odds of CGRA**	**95% CI**	*** P***	No.	%	No.	%	No.	%	No.	%
TCN vs. Targeted Print	3.129	2.028, 4.827	<.0001	191 of 641	33.5	92 of 212	53.2	53 of 216	26.7	46 of 213	23.1
TCN vs. Usual Care	3.778	2.422, 5.894	<.0001								
Targeted Print vs. Usual Care	1.207	0.766, 1.904	0.4173								

**3b. CGRA Intentions at the One-Month Follow-Up**			**CGRA Intentions = Yes**								
			**Total**		**TCN**		**Targeted Print**		**Usual Care**		**p-value**
**Study Arms Comparisons**	**Odds of CGRA**	**95% CI**	No.	%	No.	%	No.	%	No.	%	<.0001
TCN vs. Targeted Print	3.129	(2.028, 4.827)	191	33.5	92	53.2	53	26.6	46	23.1	
TCN vs. Usual Care	3.778	(2.422, 5.894)									
Targeted Print vs. Usual Care	1.207	(0.766, 1.904)									
*Percentages are based on non-missing counts

**Table 4.** Within and between study arm comparisons using mixed model analysis.

Within study arms							Between study arms			
Variables	Study Arms	Baseline Mean (se)	One-Month Mean (se)	Mean Diff. (se)	95% CI	Cohen’s *d*	Study Arms	Mean (se)	95% CI	Cohen’s *d*
**CGRA Intentions**	**TCN**	2.56 (0.08)	3.44 (0.09)	**0**.874***	**(0.639, 1.110)**	**0**.**76**	**TCN vs. UC**	**0.694** (0.167)***	**(0.367, 1.021)**	**0**.**62**
**CGRA Intentions**	**TP**	2.56 (0.08)	2.80 (.0.08)	**0**.232*	**(0.005, 0.459)**	**0**.**26**	**TCN vs. TP**	**0.642** (0.167)***	**(0.315, 0.969)**	**0**.**64**
**CGRA Intentions**	UC	2.51 (0.08)	2.69 (0.08)	0.180	(−0.046, 0.406)	0.23	TP vs. UC	0.052 (0.163)	(−0.268, 0.372)	0.03
**Perceived** **Susceptibility**	**TCN**	13.28 (0.23)	14.05 (0.25)	**0**.773*	**(0.109, 1.437)**	**0**.**25**	**TCN vs. UC**	**0.741** (0.473)*	(−0.185, 1.668)	**0**.**24**
Perceived Susceptibility	TP	13.00 (0.23)	13.41 (0.24)	0.41	(−0.235, 1.055)	0.13	TCN vs. TP	0.363 (0.473)	(−0.564, 1.289)	0.12
Perceived Susceptibility	UC	13.29 (0.23)	13.35 (0.24)	0.032	(−0.613, 0.677)	0.02	TP vs. UC	0.378 (0.466)	(−0.534, 1.291)	0.11
Perceived Severity	TCN	17.81 (0.17)	17.52 (0.18)	−0.386	(−0.890, 0.118)	0.20	TCN vs. UC	−0.500 (0.358)	(−1.202, 0.201)	0.22^++^
Perceived Severity	TP	17.71 (0.17)	17.53 (0.18)	−0.185	(−0.673, 0.303)	0.05	TCN vs. TP	−0.201 (0.358)	(−0.902, 0.500)	0.12
Perceived Severity	UC	17.21 (0.17)	17.32 (0.18)	0.115	(−0.373, 0.603)	0.04	TP vs. UC	−0.299 (0.352)	(−0.990 0.392)	0.09
Response Efficacy	TCN	16.82 (0.19)	16.59 (0.21)	−0.23	(−0.777, 0.317)	0.11	TCN vs. UC	−0.245 (0.197)	(−1.006, 0.517)	0.10
Response Efficacy	TP	16.35 (0.19)	16.32 (0.19)	−0.033	(−0.564, 0.498)	0.02	TCN vs. TP	−0.197 (0.389)	(−0.959, 0.564)	0.09
Response Efficacy	UC	16.41 (0.19)	16.41 (0.19)	0.015	(−0.516, 0.546)	0.01	TP vs. UC	−0.047 (0.383)	(−0.797, 0.702)	0.01
**Self-Efficacy**	**TCN**	13.57 (0.21)	14.24 (0.23)	**0**.666*	**(0.049, 1.283)**	**0**.**17**	TCN vs. UC	0.481 (0.438)	­­(−0.377 1.340)	0.13
Self-Efficacy	TP	13.11 (0.21)	13.27 (0.22)	0.162	(−0.436, 0.760)	0.05	TCN vs. TP	0.504 (0.438)	(−0.356, 1.363)	0.14
Self-Efficacy	UC	13.40 (0.21)	13.58 (0.22)	0.184	(−0.414, 0.782)	0.06	TP vs. UC	−0.022 (0.432)	(−0.868, 0.824)	0.01
HBOC Knowledge	TCN	5.68 (0.19)	6.16 (0.19)	0.484	(−0.037, 1.005)	0.15	TCN vs. UC	0.535 (0.376)	­­(−0.201, 1.271)	0.18
HBOC Knowledge	TP	5.61 (0.19)	5.99 (0.19)	0.374	(−0.143, 0.891)	0.13	TCN vs. TP	0.109 (0.375)	(−0.625, 0.844)	0.04
HBOC Knowledge	UC	5.90 (0.19)	5.85 (0.19)	−0.051	(−0.572, 0.470)	0.02	TP vs. UC	0.425 (0.375)	(−0.309, 1.159)	0.16
Fear of HBOC	TCN	2.44 (0.06)	2.53 (0.06)	0.086	(−0.085, 0.257)	0.13	TCN vs. UC	0.038 (0.122)	­­(−0.200, 0.277)	0.05
Fear of HBOC	TP	2.34 (0.06)	2.49 (0.06)	0.149	(−0.018, 0.316)	0.22^+^	TCN vs. TP	−0.063 (0.122)	(−0.302, 0.176)	0.10
Fear of HBOC	UC	2.40 (0.06)	2.45 (0.06)	0.047	(−0.120, 0.214)	0.08	TP vs. UC	0.101 (0.120)	(−0.134, 0.337)	0.15
CW–Frequency	TCN	2.10 (0.07)	2.07 (0.08)	−0.036	(−0.248, 0.176)	0.04	TCN vs. UC	−0.081 (0.150)	­­(−0.376, 0.213)	0.09
CW–Frequency	TP	2.09 (0.07)	2.05 (0.08)	−0.043	(−0.249, 0.163)	0.03	TCN vs. TP	0.007 (0.150)	(−0.287, 0.302)	0.01
CW–Frequency	UC	2.12 (0.07)	2.17 (0.07)	0.045	(−0.161, 0.251)	0.05	TP vs. UC	−0.088 (0.148)	(−0.378, 0.201)	0.08
CW–Intensity	TCN	4.18 (0.14)	4.02 (0.14)	−0.153	(−0.549, 0.243)	0.12	TCN vs. UC	−0.178 (0.281)	­­(−0.730, 0.373)	0.17
CW–Intensity	TP	3.92 (0.14)	4.20 (0.14)	0.287	(−0.097, 0.671)	0.22^+^	TCN vs. TP	−0.441 (0.281)	(−0.992, 0.111)	0.34^++^
CW–Intensity	UC	4.29 (0.14)	4.33 (0.14)	0.025	(−0.359, 0.409)	0.04	TP vs. UC	0.263 (0.277)	(−0.280, 0.805)	0.18
Fatalism and Destiny	TCN	7.37 (0.16)	7.49 (0.17)	0.119	(−0.338, 0.576)	0.08	TCN vs. UC	−0.024 (0.325)	­­(−0.661, 0.613)	0.05
Fatalism and Destiny	TP	7.70 (0.16)	7.76 (0.16)	0.054	(−0.389, 0.497)	0.04	TCN vs. TP	0.065 (0.325)	(−0.572, 0.701)	0.03
Fatalism and Destiny	UC	7.44 (0.16)	7.59 (0.16)	0.143	(−0.300, 0.586)	0.13	TP vs. UC	−0.089 (0.320)	(−0.715, 0.538)	0.08

Note: UC = Usual Care, TP = Targeted Print.

*p < 0.05.

**p < 0.01.

***p < 0.001.

**Figure 3.**
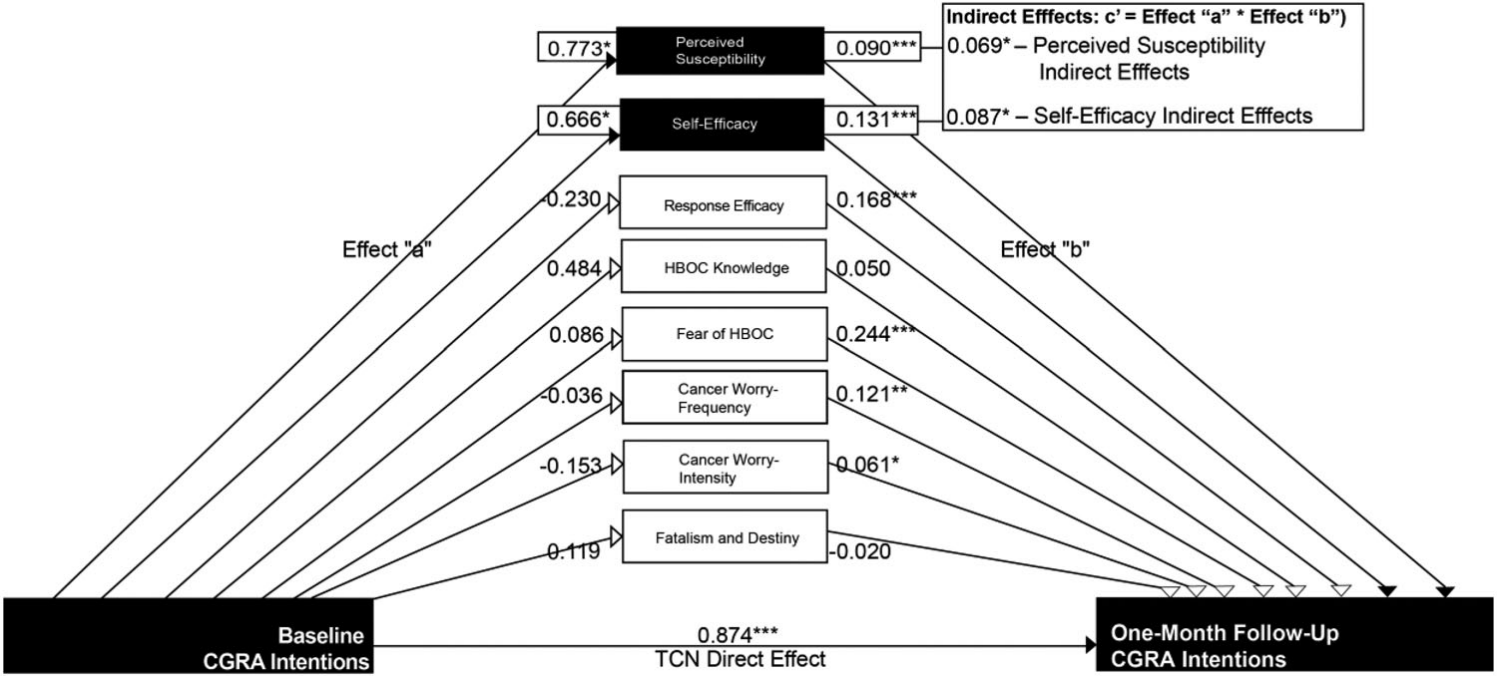
Single mediation analysis, indirect effects of theorized mediators on TCN, baseline to the one-month follow-up. Notes: Figure 3 illustrates direct and indirect mediation effects of the theorized mediation variables on TCN. Two variables, perceived susceptibility and self-efficacy significantly mediated the relationship between TCN and CGRA intentions. *p < 0.05, **p < 0.01, ***p < 0.001.

While mediation analysis produced no significant pre-to-post between-group changes in CGRA intentions (see Supplemental Statistical Methods and Table 1), we performed mediation analysis in the TCN arm to understand the mechanisms underlying improvements in CGRA intentions from baseline to the one-month follow-up. We found that significant within-group mediation occurred in TCN, shown in Table 5 and Figure 3. We did not find significant within-group mediation, however, for usual care (Table 6) or targeted print (Table 7).

**Table 5.** Single mediation analysis: indirect effects of theorized mediation variables on TCN.

TCN	Effect ‘a’	95%CI	Effect ‘b’	95% CI	Indirect Effect	95%CI	%PM
**Perceived Susceptibility**	**0**.773*	**(0.122, 1.424)**	**0**.090***	**(0.055, 0.125)**	**0**.069*	**0.010, 0.141**	7.9%
Perceived Severity	−0.386	(−0.837, 0.065)	0.075***	(0.024, 0.126)	−0.029	−0.077, 0.005	−3.3%
**Response Efficacy**	**0**.666*	**(0.080, 1.252)**	**0**.131***	**(0.094, 0.168)**	**0**.087*	**0.010, 0.174**	10.0%
Self-Efficacy	−0.230	(−0.730, 0.270)	0.168***	(−0.123, 0.213)	−0.039	−0.127, 0.045	−4.5%
HBOC Knowledge	0.484	(−0.075, 1.043)	0.050	(−0.001, 0.101)	0.024	−0.005, 0.072	2.7%
Fear of Hereditary Breast and Ovarian Cancer	0.086	(−0.085, 0.257)	0.244***	(0.109, 0.379)	0.021	−0.021, 0.071	2.4%
Cancer Worry–Frequency	−0.036	(−0.250, 0.178)	0.121**	(0.009, 0.233)	−0.004	−0.037, 0.024	−0.5%
Cancer Worry–Intensity	−0.153	(−0.549, 0.243)	0.061*	(0.000, 0.122)	−0.009	−0.043, 0.016	−1.0%
Fatalism and Destiny	0.119	(−0.353, 0.591)	−0.020	(−0.069, 0.029)	−0.002	−0.023, 0.013	−0.2%
**CGRA Intentions**	**TCN Direct Effect**	**95% CI**					
	**0**.874***	**(0.635, 1.113)**					

Note: %PM = Percent mediation effects contribute to the direct effect.

*p < 0.05.

**p < 0.01.

***p < 0.001.

**Table 6.** Single mediation analysis: indirect effects of theorized mediation variables on usual care.

TCN	Effect ‘a’	95% CI	Effect ‘b’	95% CI	Indirect Effect	95% CI	% PM
Perceived Susceptibility	0.032	(−0.623, 0.686)	0.118	(0.088, 0.149)	0.004	(−0.075, 0.083)	2.08%
Perceived Severity	0.115	(−0.413, 0.642)	0.013	(−0.028, 0.054)	0.002	(−0.012, 0.019)	0.83%
Response Efficacy	0.015	(−0.556, 0.585)	0.138	(0.102, 0.173)	0.002	(−0.078, 0.082)	1.11%
Self-Efficacy	0.184	(−0.411, 0.78)	0.13	(0.095, 0.164)	0.024	(−0.054, 0.104)	13.25%
HBOC Knowledge	−0.051	(−0.554, 0.452)	−0.013	(−0.058, 0.032)	0.001	(−0.013, 0.016)	0.36%
Fear of Hereditary Breast and Ovarian Cancer	0.047	(−0.117, 0.212)	0.434	(0.309, 0.558)	0.021	(−0.051. 0.095)	11.40%
Cancer Worry–Frequency	0.045	(−0.159, 0.25)	0.148	(0.044, 0.252)	0.007	(−0.025, 0.043)	3.74%
Cancer Worry–Intensity	0.025	(−0.369, 0.419)	0.086	(0.032. 0.14)	0.002	(−0.034, 0.04)	1.20%
Fatalism and Destiny	0.143	(−0.283, 0.569)	0.023	(−0.028, 0.073)	0.003	(−0.011, 0.024)	1.79%
**CGRA Intentions**	**UC Direct Effect**	**95% CI**					
	0.18	(−0.045, 0.405)

**Table 7.** Single mediation analysis: indirect effects of theorized mediation variables on targeted print.

TP	Effect ‘a’	95% CI	Effect ‘b’	95% CI	Indirect Effect	95% CI	% PM
Perceived Susceptibility	0.41	(−0.239, 1.059)	0.099	(0.067, 0.13)	0.04	(−0.023, 0.11)	17.42%
Perceived Severity	−0.185	(−0.678, 0.309)	0.01	(−0.034, 0.053)	−0.002	(−0.02, 0.012)	0.77%
Response Efficacy	−0.033	(−0.563, 0.497)	0.131	(0.093, 0.17)	−0.004	(−0.076, 0.066)	1.86%
Self-Efficacy	0.162	(−0.465, 0.789)	0.14	(0.109, 0.172)	0.023	(−0.066, 0.113)	9.78%
HBOC Knowledge	0.374	(−0.121, 0.87)	0.064	(0.018, 0.11)	0.024	(−0.007, 0.068)	10.30%
Fear of Hereditary Breast and Ovarian Cancer	0.149	(−0.019, 0.317)	0.371	(0.248, 0.493)	0.055	(−0.007, 0.125)	23.74%
Cancer Worry–Frequency	−0.043	(−0.247, 0.161)	0.183	(0.079, 0.287)	−0.008	(−0.05, 0.031)	3.38%
Cancer Worry–Intensity	0.287	(−0.087, 0.662)	0.076	(0.019, 0.134)	0.022	(−0.006, 0.063)	9.45%
Fatalism and Destiny	0.054	(−0.393, 0.501)	−0.028	(−0.076, 0.02)	−0.002	(−0.022, 0.016)	0.66%
**CGRA Intentions**	**TP Direct Effect**	**95% CI**					
	0.232*	(0.008, 0.456)					

Note: %PM = Percent mediation effects contribute to the direct effect.

*p < 0.05.

**p < 0.01.

***p < 0.001.

Single variable mediation analysis indicated significant indirect effects in TCN for perceived susceptibility (0.069, CI: 0.010, 0.141) and self-efficacy (0.087, CI: 0.010, 0.174), which accounted for 7.9% and 10.0% of TCN’s direct effects, respectively. Our multivariable mediation analysis, reported in Table 8, indicated that the indirect effects of perceived susceptibility (0.056, CI: 0.008, 0.118) and self-efficacy (0.077, CI: 0.009, 0.156) remained significant, though their contribution to TCN’s direct effects (6.4% and 8.8%, respectively) attenuated slightly.

**Table 8.** Multivariable mediation analysis for TCN.

TCN	Effect ‘a’	95%CI	Effect ‘b’	95%CI	Indirect Effects	95%CI	%PM
Perceived Susceptibility	0.072***	(0.037, 0.107)	0.773*	(0.122, 1.424)	0.056*	(0.008, 0.118)	6.4%
Self-Efficacy	0.116***	(0.079, 0.153)	0.666*	(0.080, 1.252)	0.077*	(0.009, 0.156)	8.8%
**CGRA Intentions**	**TCN Direct Effect**	**95% CI**					
	0.874***	(0.635, 1.113)					

Note: %PM = Percent mediation effects contribute to the direct effect.

*p < 0.05.

**p < 0.01.

***p < 0.001.

## Discussion

Our results indicated that a risk-based psychoeducational intervention delivered by telephone could increase behavioral intentions to obtain CGRA within a short timeframe (one month). To our knowledge, this is the first population-based study using a theoretically grounded, remote behavioral intervention to motivate guideline-based CGRA uptake among breast and ovarian cancer survivors at increased risk for HBOC. The intervention significantly improved CGRA intentions from baseline to one month. TCN’s motivational session had a much greater direct effect on CGRA intentions (0.874, *p* < .001) than the targeted print brochure (0.232, *p* < .045). Large direct effects can make it difficult to determine the indirect effects of mediators on the outcome, such as the theoretical variables identified in this study (Acharya et al., 2016). This may have played a role in the lack of observed between-group mediation. Our within-group mediation analysis for TCN indicated mediation by both perceived susceptibility and self-efficacy. This finding partially supported our hypothesis that our theorized mediators (perceived susceptibility, self-efficacy, response efficacy, HBOC knowledge, fear of HBOC, cancer worry, fatalism, and destiny) mediated the relationship between TCN and CGRA intentions.

The results aligned with previous studies that found tailored risk messaging increases perceived susceptibility, which we observed in TCN (Anderson-Lewis et al., 2018; Rees et al., 2018; Steffen et al., 2015). During the TCN session, health educators leveraged participants’ baseline survey responses and real-time interactions to explore their perceived HBOC risk and efficacy beliefs and implementation intentions. These interactions helped cast coaches as trusted informational resources, well positioned to encourage increases in CGRA intentions (Carpenter & Sherbino, 2010; Gollwitzer, 1996; Spencer & Wheeler, 2016). The coaches’ clear, personalized guidance also may have contributed to increases in self-efficacy – a factor that may be key to translating CGRA intentions into uptake. Past research has shown that individuals with high self-efficacy are better equipped to navigate health services when presented with a health risk (Gollwitzer, 1996) and avoid maladaptive approaches to controlling fear (Witte, 1992). As expected, perceived severity was not a significant mediator of CGRA intentions due to nearly universal perceptions of cancer’s potential harm.

However, we had anticipated improvements in more of our theorized mediators, notably response efficacy, which has been found in other studies to be a strong mediator of behavioral intention (Brumbach et al., 2017; Gollwitzer, 1996). This may reflect our study population, many of whom had extensive experience with cancer treatment. They perhaps already understood the importance of cancer screening and were more readily convinced of genetic counseling and testing’s potential benefits for themselves and their family members. Past studies indicate that cancer survivors are more likely to engage in cancer surveillance and prevention than those who have never had cancer (Trask et al., 2005).

The literature also indicates that individuals who have undergone cancer treatment may remain fearful of cancer and its possible recurrence, potentially explaining why fear of HBOC, cancer worry, fatalism, and destiny failed to mediate the CGRA intentions (Meissner et al., 2021; Takeuchi et al., 2020). These emotions may be difficult to change with a short-term intervention like TCN. Treatment experience, which perhaps provided participants access to cancer information, also may explain why HBOC knowledge failed to mediate CGRA intentions (Gollwitzer, 1996; Takeuchi et al., 2020).

GRACE had multiple strengths, including its three-arm, randomized superiority trial design, which provided insight into how TCN performed against a targeted print brochure and usual care. Our study also leveraged an integrated theorized framework that informed the study design and, in the TCN intervention, helped bridge the gap between motivation and behavioral intentions among participants. GRACE leveraged state cancer registries to identify and engage breast and ovarian cancer survivors who otherwise might not have been reached and/or may have deferred cancer risk assessment and genetic counseling and testing (Millar et al., 2019). Nearly 80% of individuals who contacted and found to be eligible actually enrolled in the study. GRACE tested a unique intervention, TCN, designed to increase awareness about the availability of cancer genetic risk counseling, motivate its use, and navigate women to these health services. The intervention’s tailored, theoretically guided psychoeducational intervention, facilitated by a community health educator, differed from genetic counseling, which is a clinical service to inform patients about the benefits, risks, and limitations of genetic testing, preparing them for possible test results and medical recommendations. It is intended to help patients make informed decisions, not motivate genetic testing.

The study had several limitations, including its focus on CGRA intentions rather than behavior. While CGRA uptake is a stronger outcome, this study encompassed a brief, one-month timeframe; participants would have had little opportunity to obtain genetic counseling and/or testing before the one-month follow-up. We also did not observe significant pre-to-post between-group changes in CGRA intentions in our mediation analysis. As noted earlier, this may reflect the large direct effects of the TCN intervention on CGRA intentions, which may inhibit the observation of identified mediators’ indirect effects (Acharya et al., 2016). Some researchers argue that failing to account for all possible intermediate variables in a mediation identification can introduce selection and intermediate variable bias (a type of posttreatment bias) (Imai & Yamamoto, 2013).

We leveraged standard approaches to mediation analysis in this study, which assume no confounding among mediatiors (Acharya et al., 2016). Study results reflected the results of our direct effects analysis, which found that TCN had a significantly larger effect on CGRA intentions than the targeted print brochure. Our between-group mediation analyses were null; however, within-group mediation analysis of the TCN arm identified two significant mediators, perceived susceptibility and self-efficacy. These results offered insight into mechanisms underlying pre-to-post improvements in CGRA intentions from baseline to the one-month follow-up. Additionally, while our study had a large, diverse sample (Hispanic participants 27%, rural dwellers 17%), Black and other race populations were not optimally represented, thereby limiting the generalizability of the findings. These results underscore the critical need to address the underrepresentation of Black persons in research and the underutilization of cancer genetic services (Hann et al., 2017; Sutton et al., 2019).

Our findings have implications for the use of theory-based psychoeducational behavioral motivation interventions in genomic medicine. The TCN intervention increased motivation to have a CGRA, demonstrating that a tailored, psychoeducational intervention could be successfully delivered remotely to women at increased HBOC risk. While not a replacement for genetic counseling, GRACE’s communication strategies could be incorporated into recommendations delivered by case managers and health educators at the point-of-care in the clinic, or remotely as a telehealth visit to motivate CGRA uptake. This approach may benefit clinics with limited budgets and staff seeking a low-cost, expedient method to alert women of an increased HBOC risk, educate them and help them make decisions about seeking genetic counseling and testing, and link them to CGRA clinical services. While the original motivational session was less than an hour, streamlining it to 15 min or less could make the TCN intervention even more scalable. Health systems and practitioners who incorporate elements of GRACE into their routine care procedures may tailor the intervention to their patients’ cancer history and personal circumstances. This personalization may be especially helpful for individuals from underserved populations who otherwise might not engage in CGRA due to limited access and psychosocial barriers, such as feelings of medical mistrust (Gómez-Trillos et al., 2020; Komenaka et al., 2016; Sutton et al., 2019).

The intervention’s telephone delivery helped ensure participants across ethnic groups, socioeconomic strata, literacy levels, and technological acumen and access could participate in TCN (Kinney et al., 2016; Peshkin et al., 2016). While most participants completed their surveys online, approximately one-third opted to complete them by telephone. The interactions between study staff and participants could have introduced response bias, with participants answering questions based on perceived desires of the researcher (Acharya et al., 2016). However, providing the option to complete the surveys by telephone enabled all participants to complete their assessments according to their preference.

The results speak to the feedback of participants in the formative research for the GRACE Study, who stressed that a telephone-based intervention provided more accessible and immediate engagement with a health coach beyond that afforded through a brochure (Kinney et al., 2018). Such flexibility proved essential to ensuring participants’ engagement with GRACE during the COVID-19 pandemic. An in-depth analysis of audio files of the TCN sessions may help identify additional theoretical mechanisms underlying its effects.

## Data Availability

For information about the GRACE data set, please email the study team at ak1617@sph.rutgers.edu.
